# Identification of natural inhibitors of *Entamoeba histolytica* cysteine synthase from microbial secondary metabolites

**DOI:** 10.3389/fmicb.2015.00962

**Published:** 2015-09-14

**Authors:** Mihoko Mori, Ghulam Jeelani, Yui Masuda, Kazunari Sakai, Kumiko Tsukui, Danang Waluyo, Tarwadi, Yoshio Watanabe, Kenichi Nonaka, Atsuko Matsumoto, Satoshi Ōmura, Tomoyoshi Nozaki, Kazuro Shiomi

**Affiliations:** ^1^Kitasato Institute for Life Sciences, Kitasato UniversityTokyo, Japan; ^2^Graduate School of Infection Control Sciences, Kitasato UniversityTokyo, Japan; ^3^Department of Parasitology, National Institute of Infectious DiseasesTokyo, Japan; ^4^Biotech Center, Badan Pengkajian Dan Penerapan TeknologiBanten, Indonesia; ^5^Research and Development Division, MicroBiopharm Japan Co. LtdIwata, Japan; ^6^Graduate School of Life and Environmental Sciences, University of TsukubaTsukuba, Japan

**Keywords:** amebiasis, compound library screening, cysteine synthase, *Entamoeba histolytica*, secondary metabolites

## Abstract

Amebiasis is a common worldwide diarrheal disease, caused by the protozoan parasite, *Entamoeba histolytica*. Metronidazole has been a drug of choice against amebiasis for decades despite its known side effects and low efficacy against asymptomatic cyst carriers. *E. histolytica* is also capable of surviving sub-therapeutic levels of metronidazole *in vitro*. Novel drugs with different mode of action are therefore urgently needed. The sulfur assimilatory *de novo* L-cysteine biosynthetic pathway is essential for various cellular activities, including the proliferation and anti-oxidative defense of *E. histolytica*. Since the pathway, consisting of two reactions catalyzed by serine acetyltransferase (SAT) and cysteine synthase (CS, *O*-acetylserine sulfhydrylase), does not exist in humans, it is a rational drug target against amebiasis. To discover inhibitors against the CS of *E. histolytica* (EhCS), the compounds of Kitasato Natural Products Library were screened against two recombinant CS isozymes: EhCS1 and EhCS3. Nine compounds inhibited EhCS1 and EhCS3 with IC_50_ values of 0.31–490 μM. Of those, seven compounds share a naphthoquinone moiety, indicating the structural importance of the moiety for binding to the active site of EhCS1 and EhCS3. We further screened >9,000 microbial broths for CS inhibition and purified two compounds, xanthofulvin and exophillic acid from fungal broths. Xanthofulvin inhibited EhCS1 and EhCS3. Exophillic acid showed high selectivity against EhCS1, but exhibited no inhibition against EhCS3. *In vitro* anti-amebic activity of the 11 EhCS inhibitors was also examined. Deacetylkinamycin C and nanaomycin A showed more potent amebicidal activity with IC_50_ values of 18 and 0.8 μM, respectively, in the cysteine deprived conditions. The differential sensitivity of trophozoites against deacetylkinamycin C in the presence or absence of L-cysteine in the medium and the IC_50_ values against EhCS suggest the amebicidal effect of deacetylkinamycin C is due to CS inhibition.

## Introduction

Amebiasis is a common diarrheal disease in humans, arising from infection with the parasitic protozoan *Entamoeba histolytica*. WHO estimates 50 million people are infected worldwide, resulting in 40,000–100,000 deaths annually (Harque et al., 2003; Stanley, 2003; Ximénez et al., 2009). Transmission occurs via the fecal–oral route, either directly by person to person contact or indirectly through consumption of contaminated food or water. In developed countries, including Japan, domestic cases of amebiasis are increasing among men who have sex with men, particularly those infected with HIV (Watanabe et al., 2011; Hung et al., 2012).

Metronidazole, has been a drug of choice against amebiasis for decades despite its low efficacy against asymptomatic cyst carriers (Ali and Nozaki, 2007). Moreover, metronidazole is teratogenic and causes several adverse side effects, such as nausea, vomiting, and a metallic taste (Ohnishi et al., 2014). It has been shown that *E. histolytica* is capable of surviving sub-therapeutic levels of metronidazole *in vitro* (Samarawickrema et al., 1997; Wassmann et al., 1999). Therefore, new drugs with targets and modes of action different from those of metronidazole are urgently needed.

**FIGURE 1:**
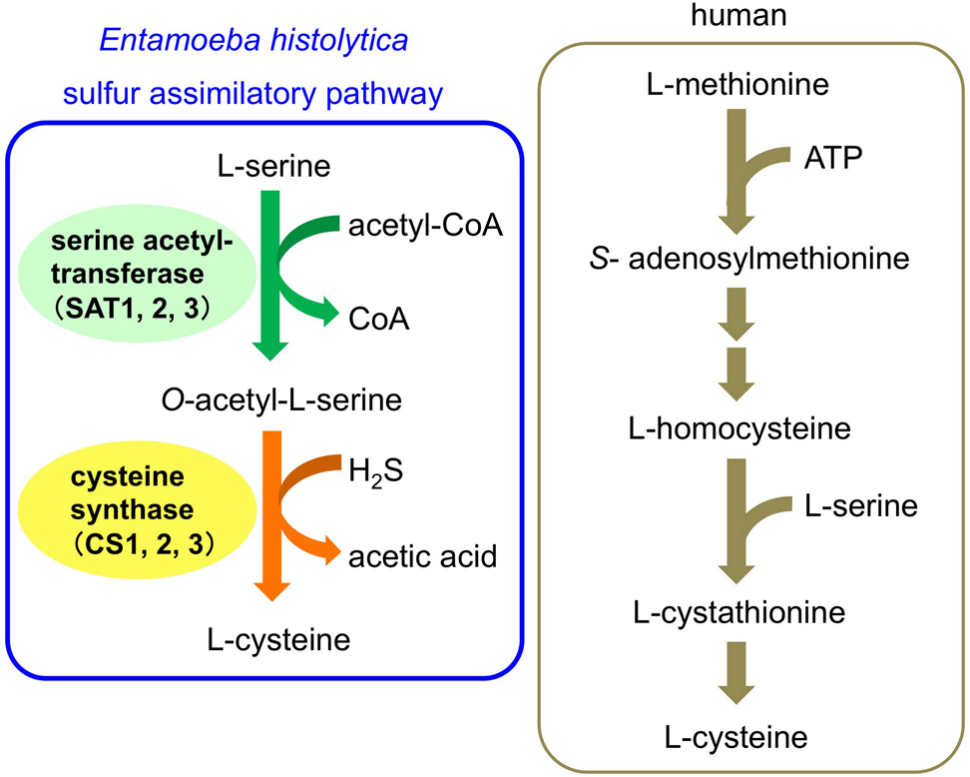
**Cysteine biosynthetic pathway of *Entamoeba histolytica* and human**.

The *de novo* biosynthetic pathway of L-cysteine, is essential for various cellular activities, including the attachment, motility, proliferation, and anti-oxidative defense of *E. histolytica* (Gillin and Diamond, 1981; Fahey et al., 1984; Jeelani et al., 2010, 2014; Hussain et al., 2011). Since the homologous pathway does not exist in humans, it could be a rational drug target for anti-amebic agents. This pathway consists of two reactions catalyzed by serine acetyltransferase (SAT, EC 2.3.1.30) and cysteine synthase (CS, *O*-acetylserine sulfhydrylase, EC 2.5.1.47) (**Figure 1**; Nozaki et al., 1998, 1999,  2005). In the first reaction, L-serine is acetylated with acetyl-CoA by SAT, and in the second reaction, the alanyl moiety of *O*-acetylserine is transferred to sulfide by CS to produce L-cysteine . This sulfur-assimilatory cysteine biosynthetic pathway exists in bacteria, plants, and some parasitic protozoa, including *Leishmania major*, *Trichomonas vaginalis*, and *Trypanosoma cruzi*. The *E. histolytica* genome encodes three isotypes of SAT (EhSAT1-3) and CS (EhCS1-3) (Nozaki et al., 2005; Ali and Nozaki, 2007; Hussain et al., 2009). The SAT and CS enzymes of *E. histolytica* have unique features. Three isotypes of SAT and CS are all present in the cytosol, whereas in plants three isotypes are compartmentalized, i.e., the cytosol, mitochondria, and plastids. EhCS1 and EhSAT1 do not form a heteromeric complex (Nozaki et al., 1999; Kumar et al., 2011), which is in stark contrast to bacteria and plants, in which SAT and CS form such a complex (Kredich et al., 1969; Feldman-Salit et al., 2009). In addition, EhSAT1-3 are both sequence-wise and biochemically divergent; EhSAT1-3 show remarkably different sensitivity against allosteric feedback by L-cysteine (Ali and Nozaki, 2007; Hussain et al., 2009). Thus, amebic SATs and EhCSs are a rational target to develop anti-amebic drugs.

The sulfur-assimilatory cysteine biosynthetic pathway in plants and bacteria have been exploited for drug development (Salsi et al., 2010; Amori et al., 2012; Spyrakis et al., 2013). Nagpal et al. (2012) recently performed *in silico* screening of ZINC database studies based on the crystal structure of EhCS1. One compound was discovered and showed moderate inhibitory activity against EhCS1 (74% inhibition at 100 μM) *in vitro*. A similar virtual screening was also carried out against *Trichomonas* CS, which yielded some potential inhibitors (Singh et al., 2013).

Structure-based drug discovery can identify potential ligand compounds fit for predicted active sites of target proteins. Alternatively, screening of natural product libraries or microbial extracts by directly evaluating inhibitory activity against target enzymes can also identify existent and novel inhibitors. Consequently, we developed an enzymatic assay system using recombinant CSs to identify inhibitors with potentially novel and diverse structures.

Natural products have been playing a very important role in drug discovery and development for over a century. In particular, microbial secondary metabolites have provided many kinds of compounds, and it is widely considered that a plethora of unique chemical compounds must still exist undiscovered in the microbial environment (Newman and Cragg, 2012).

Several anti-amebic compounds have been found from natural sources, particularly from plants. However, microbial metabolite sources have not been vigorously exploited (Singh et al., 2009) as plants or herbs used for traditional remedies have been historically utilized to isolate active anti-amebic components.

In this study, we utilized two libraries of microbial secondary metabolites, a chemically defined natural product library and microbial culture broths as a source of novel lead compounds for anti-amebic drugs. We first screened 316 compounds from the Kitasato Natural Products Library against the recombinant enzymes of two representative CSs: EhCS1 and EhCS3. We also screened extracts from more than 9,000 fungal and actinomycete culture broths against EhCS1 and EhCS3. We obtained two compounds, xanthofulvin and exophillic acid, as EhCS1 inhibitors from the broths of two fungi, *Penicillium* sp. and *Exophiala* sp., respectively. Xanthofulvin, which contains a xanthone moiety in its structure, inhibited both EhCS1 and EhCS3. The other inhibitor, exophillic acid, showed high selectivity against EhCS1. *In vitro* anti-amebic activity of the 11 EhCS inhibitors found were also evaluated.

## Materials and Methods

### Expression and Purification of Recombinant *E. histolytica* CSs

The *E. coli* BL21 (DE3) cell introduced pET-EhCS (CS1, CS3) construct was used for protein expression. Each EhCSs protein was expressed by 1 mM isopropyl β-D-thiogalactopyranoside induction for 12 h at 30°C. After induction, the *E. coli* cell was harvested and re-suspended in the lysis buffer (50 mM Tris-HCl, pH 8.0, and 300 mM NaCl) containing 0.5 mM phenylmethylsulfonyl fluoride. The suspension was sonicated and centrifuged at 24,000 ×*g* for 30 min at 4°C. The histidine-tagged rEhCS proteins were purified from the supernatant fraction using a Ni^2+^-NTA column and tagged proteins were eluted with 100–200 mM imidazole in 50 mM Tris-HCl, pH 8.0, and 300 mM NaCl. The purity of the eluted rEhCS proteins was confirmed by SDS-PAGE and then dialyzed in 50 mM Tris-HCl, and 150 mM NaCl, pH 8.0 containing 10% glycerol (v/v) for 24 h at 4°C. The dialyzed proteins were stored at–80°C with glycerol in small aliquots until use, purified proteins retaining activity for more than three months when stored at this temperature.

#### Evaluation of Enzymatic Activities

For measurement of CS inhibitory activity, 10 μl of sample solution [dimethylsulfoxide (DMSO)/H_2_O = 1/1 solution] and 30 μl of reaction mixture, composed of 2 μl of 50 mM *O*-acetyL-L-serine, 2 μl of 50 mM Na_2_S, 12.5 μl of 200 mM Tris-HCl (pH 7.5), and 13.5 μl of water, was poured into each well of a flat-bottom 96-well plate. After 10 μl of enzyme solution (containing 0.1 μg of rEhCS1 or rEhCS3 in H_2_O) was added to each well, plates were incubated for 15 min at 37°C. The final concentrations of reagents in each well are; 2 mM *O*-acetyL-L-serine, 2 mM Na_2_S, and 50 mM Tris-HCl. The enzymatic reaction was stopped by adding 50 μl of acetic acid (>99.9%), and subsequently the reaction for cysteine detection was started by adding 50 μl of acid-ninhydrin reagent (Gaitonde, 1967). The mixture in each well was transferred to a 96-well PCR plate and heated at 95°C for 10 min. After the plate was cooled on ice, the mixture was transferred into a flat-bottom 96-well plate filled with 100 μl of ethanol (EtOH). Absorbance at 560 nm was measured by spectrophotometer (SH-9000Lab, Corona Electric, Ibaraki, Japan). A standard inhibitor of this assay system does not exist, therefore we defined that the values without samples are 0% inhibition, and the values without both samples and enzyme are 100% inhibition. To determine whether the sample interferes the acid-ninhydrin reagent reaction, 10 μl of sample solution, 10 μl of water, and 30 μl of 1 mM cysteine solution (cysteine hydrochloric acid salt in water) were mixed in a well of 96-well microtiter plate, and then 50 μl of acetic acid (>99.9%) and 50 μl of acid-ninhydrin reagent were added. The mixture was treated and measured the absorbance at 560 nm as above mentioned to check the effect of the sample to the color reaction.

#### Evaluation of Cytotoxicity against MRC-5 Cells

Human fibroblast cells, MRC-5, were plated on 96-well flat bottom plates at a density of 1.5 × 10^4^ cells/well with 100 μl of MEM medium (Life Technologies, Grand Islands, NY, USA) containing 10% fetal bovine serum (Hana-nesco Bio, Tokyo, Japan) and 1% penicillin-streptomycin (Life Technologies) and incubated at 37°C with 5% CO_2_ for 2 days. Test compounds in 5 μl of 50% DMSO–H_2_O and 100 μl of MEM medium were mixed and added to each well. After 2 days cultivation at 37°C with 5% CO_2_, cell density and morphological changes were observed under a microscope. After observation, 10 μl of WST-8 solution (Dojindo, Kumamoto, Japan) was added to the cells and the plate was incubated at 37°C with 5% CO_2_ for 2 h. Then, absorbance at 450 nm was measured by spectrophotometer (SH-9000Lab, Corona Electric). Cytotoxicity was measured in duplicate. Staurosporine (our product, in Kitasato Natural Products Library) was used as a positive (cytotoxic) compound. The concentration range of test compounds are: 1.6 to 50 μg/ml (final concentration) for kerriamycin B, kerriamycin C, and aggreticin; 0.075 to 10 μg/ml for deacetylkinamycin C, deoxyfrenolicin, nanaomycin A, and naphthacemycin A_9_; 3.1 to 200 μg/ml for tetracycline and patulin; 9.4 to 75 μg/ml for exophillic acid. IC_50_ values were calculated by the equation described in the reference (Arita-Morioka et al., 2015).

#### Screening Sources

The Kitasato Natural Products Library comprises 316 compounds. Each compound was dissolved in DMSO at 1.0 mg/ml. The source microbes for screening were collected in Japan and Indonesia. Fungal strains originating in Japan were isolated from soil samples collected near plants. Actinomycetes originating in Japan were isolated from plants as well as from soil samples attached to plant roots. The Indonesian fungi and actinomycetes were isolated from soils, plants and insects. In total, 9,173 broth extract samples (4,800 fungal plus 4,373 from actinomycete) were prepared. After cultivation, the same amount of EtOH was added to each broth, which were then centrifuged. Obtained supernatants were used for screening as broth extract samples.

#### Screening of CS Inhibitors from Natural Compounds and Microbial Broths

Each 5 μl of DMSO solution (1 mg/ml, final concentration 100 μg/ml) of 316 library compounds and 5 μl of water (total 10 μl of DMSO/H_2_O = 1/1 solution per well) were poured into each well of a 96-well microtiter plate to screen for CS inhibitors. For screening microbial broths, 10 μl of the individual broth extract samples (50% EtOH solution) were poured into each well of a 96-well microtiter plate and dried up *in vacuo*. After drying, 10 μl of DMSO/H_2_O = 1/1 solution was added and dissolved. Samples showing more than 50% inhibitory activity and having no inhibitory activities against acid ninhydrin reaction were selected for further evaluation. The inhibition values were measured in duplicate or triplicate. The concentration range of test compounds are: 0.15 to 20 μg/ml (final concentration) for kerriamycin B, kerriamycin C, and aggreticin; 2.0 to 250 μg/ml for deacetylkinamycin C, deoxyfrenolicin, nanaomycin A, tetracycline and patulin; 1.5 to 200 μg/ml for naphthacemycin A_9_; 3.1 to 400 μg/ml for xanthofulvin, terreinol and citromycetin; 0.06 to 200 μg/ml for exophillic acid. IC_50_ values were calculated as shown in Section “Evaluation of Cytotoxicity against MRC-5 Cells” with each concentration range.

### Isolation of CS Inhibitors from Culture Broths of Microorganisms

#### Isolation of Xanthofulvin from a Culture Broth of *Penicillium* sp. if08054

##### Producing strain and cultivation

The fungal strain *Penicillium* sp. if08054 was isolated from a soil sample collected at Jember, Java Island, Indonesia. The sequence of the 28S rDNA D1/D2 region of this strain had 100% similarity with *Penicillium lagena*. However, the results of morphological and sequencing analyses confirmed that the producing strain, if08054, was an unidentified species of the genus *Penicillium*. The medium used to culture this organism consisted of 2% rice starch, 1% glucose, 2% soybean meal, 1% KH_2_PO_4_, 0.5% MgSO_4_⋅7H_2_O (pH was not adjusted). Frozen broth of this strain (0.2 ml) was inoculated into a 250-ml Erlenmeyer flask containing 20 ml of medium and incubated in a rotary shaker (220 rpm) at 25°C for 3 days. This seed culture (0.5 ml) was inoculated into a 500-ml Erlenmeyer flask containing 50 ml of medium and the production culture was incubated in a rotary shaker (220 rpm) at 25°C for 4 days.

##### Isolation of xanthofulvin from the culture broth

The culture broth (50 ml) was extracted with 50 ml of *n*-BuOH, and the extract was evaporated and dried *in vacuo*. The dried extract (0.23 g) was dissolved in 50 ml of 50% EtOH–H_2_O, and after removing EtOH, then applied to an ODS column (30ϕ × 300 mm, YMC, Kyoto, Japan). The column was eluted with H_2_O–acetonitrile (MeCN) system to give six fractions (0, 20, 40, 60, 80, and 100% MeCN, each 0.6 l). The 40% MeCN fraction (50 mg) was purified by Sephadex LH-20 column chromatography (10ϕ × 300 mm, GE Healthcare Bio-Sciences, Piscataway, NJ, USA; solvent, MeOH). The eluate was fractionated every 1 ml. Fractions 46-50 were collected and concentrated *in vacuo* to dryness to afford xanthofulvin (1.9 mg). Fractions 41–43 contained terreinol (2.1 mg). The 20% MeCN fraction (25 mg) of ODS column chromatography was purified by preparative HPLC (column, Pegasil ODS SP100, 20ϕ × 250 mm, Senshu Scientific, Tokyo, Japan; solvent, 20% MeCN–H_2_O; flow rate, 7.0 ml/min; detection, UV at 254 nm). The peak eluted at 24–26 min was collected and concentrated *in vacuo* to dryness to afford citromycetin (7.8 mg).

Xanthofulvin. Yellowish-brown amorphous. ^1^H NMR (500 MHz, DMSO-*d_6_*) δ 7.69 (1H, s), 6.85 (1H, s), 6.49 (1H, s), 4.78 (2H, s), 2.66 (3H, s), 2.19 (3H, s). ESI-MS calcd. for C_28_H_17_O_14_ [M-H]^-^: 577.0624, found 577.0628 [M-H]^-^.

Terreinol. Yellowish-brown amorphous. ^1^H NMR (400 MHz, CD_3_OD) δ 6.97 (1H, s), 4.92 (1H, d, 16 Hz), 4.75 (1H, d, 16 Hz), 4.06 (1H, m), 3.97 (1H, dd, 15 and 7.5 Hz), 2.66 (1H, m), 2.13 (3H, s), 2.13 (1H, m, overlapped), 2.04 (1H, m), 1.89 (1H, m). ESI-MS calcd. for C_13_H_14_O_5_Na [M+Na]^+^: 273.0739, found 273.0730 [M+Na]^+^.

Citromycetin. Yellow amorphous. ^1^H NMR (400 MHz, CD_3_OD) δ 6.51 (1H, s), 6.20 (1H, s), 5.01 (2H, s), 2.33 (3H, s). ESI-MS calcd. for C_28_H_21_O_14_ [2M+H]^+^: 581.0931, found 581.0923 [2M+H]^+^.

#### Isolation of Exophillic Acid from a Culture Broth of *Exophiala* sp. FKI-7082

##### Producing strain and cultivation

The fungal strain *Exophiala* sp. FKI-7082 was isolated from a soil sample collected in Kouzu-shima Island, Japan. The producing strain FKI-7082 was classified in the genus *Exophiala* according to its morphology. This fungal strain was maintained on an LcA slant consisting of 0.1% glycerol, 0.08% KH_2_PO_4_, 0.02% K_2_HPO_4_, 0.02% MgSO_4_⋅7H_2_O, 0.02% KCl, 0.2% NaNO_3_, 0.02% yeast extract, and 1.5% agar (adjusted to pH 6.0 before sterilization). A loopful of spores was inoculated into two test tubes, each containing 10 ml of a seed medium consisting of 2% glucose, 0.5% Polypepton (Nihon Pharmaceutical, Tokyo, Japan), 0.2% yeast extract, 0.2% KH_2_PO_4_, 0.05% MgSO_4_⋅7H_2_O, and 0.1% agar (adjusted to pH 6.0 before sterilization) and incubated on a shaker at 27°C for 3 days. One milliliter of the seed culture was inoculated into each of ten 500-ml Erlenmeyer flasks, each containing 100 ml of a production medium consisting of 3% soluble starch, 1% glycerol, 2% soy bean meal, 0.3% dry yeast, 0.3% KCl, 0.05% KH_2_PO_4_, and 0.05% MgSO_4_⋅7H_2_O (adjusted to pH 6.5 before sterilization) and incubated on a rotary shaker at 27°C for 6 days.

##### Isolation of exophillic acid from the culture broth

The obtained culture broth (1 l) was extracted with 1 l of EtOH and mycelia were then removed by centrifugation and filtration. After evaporation, the residue was extracted three times with 1 l of ethyl acetate, and the organic layer was dried *in vacuo*. The dried extract (1.2 g) was dissolved in a small amount of chloroform and then applied to a silica gel column (30ϕ × 140 mm, particle size: 0.063–0.200 mm, Merck, Darmstadt, Germany). The column was eluted with chloroform–MeOH system to give five fractions (100:0, 99:1, 98:2, 95:5, 90:10, 50:50, and 0:100, each 200 ml). The two active fractions, 90:10 fraction (27 mg) and 50:50 fraction (442 mg), were combined and applied to an ODS column (30ϕ × 70 mm, YMC). An H_2_O–MeCN system (0, 20, 40, 50, 60, 80, and 100% MeCN, each 100 ml) was used as eluent. The active component was eluted in 40–100% MeCN fractions. One of the fractions, 60% MeCN fraction (151 mg), was purified by preparative HPLC (column, Pegasil ODS SP100, 20ϕ × 250 mm, Senshu Scientific; solvent, 100% MeOH; flow rate, 9.0 mL/min; detection, UV at 210 nm). The main peak was collected and concentrated *in vacuo* to dryness to afford exophillic acid (103.4 mg).

Exophillic acid. White amorphous. ^1^H NMR (400 MHz, pyridine-*d_5_*) δ 7.36 (1H, d, 2.0 Hz), 7.30 (1H, d, 2.0 Hz), 7.10 (1H, d, 2.0 Hz), 6.90 (1H, d, 2.0 Hz), 5.62 (1H, d, 7.5), 4.39 (1H, dd, 12.0, 2.5 Hz), 4.37 (1H, t, 7.5 Hz), 4.33 (1H, dd, 12.0, 5.0 Hz), 4.32 (1H, m), 4.30 (1H, m), 3.94 (1H, ddd, 7.5, 5.0, 2.5 Hz), 3.39 (1H, dt, 13.0, 7.5 Hz), 3.34 (1H, dt, 13.0, 7.5 Hz), 2.97 (2H, t, 7.5 Hz), 1.85 (2H, m), 1.76 (2H, m), 1.42 (2H, m), 1.39 (2H, m), 1.31 (4H, m), 1.23 (16H, br.s, overlapped), 0.87 (3H, t, 7.0 Hz), 0.85 (3H, t, 7.0 Hz). ESI-MS calcd. for C_38_H_55_O_12_ [M-H]^-^: 703.3694, found 703.3683 [M-H]^-^.

### Measurement of Anti-Amebic Activity

Trophozoites of *E. histolytica* clonal strain HM-1:IMSS cl6 were cultured axenically in Diamond’s BI-S-33 medium at 35.5°C (Clark and Diamond, 2002). Trophozoites were harvested in 3–4 days after inoculation of 1/30 to 1/12 of the total culture volume. After the cultures were chilled on ice for 5 min, trophozoites were collected by centrifugation at 500 ×*g* for 10 min at 4°C and washed twice with BI-S-33 medium. The obtained trophozoites were suspended in BI-S-33 medium and approximately 1 × 10^4^ trophozoites in 200 μl of BI-S-33 medium were poured into each well of a 96-well plate and incubated for 2 h. Subsequently, the medium was removed and 200 μl of BI-S-33 medium or cysteine-deprived BI-S-33 medium containing 1% (v/v) penicillin/streptomycin (Life Technologies) together with test compounds (final concentrations; 0.1, 1, 10, 100 μg/ml for kerriamycin B, kerriamycin C, aggreticin, and xanthofulvin; 0.1, 1, 10, 50, 100 μg/ml for the other compounds) was added to each well and incubated under anaerobic conditions for 48 h. After incubation, the medium was removed and 90 μl of pre-warmed Opti-MEM I (Life Technologies) and 10 μl of WST-1 solution (Dojindo) were added to each well. Viability of trophozoites was detected with absorbance at 450 nm (SH-9000Lab, Corona Electric). Cytotoxicity was measured in triplicate. IC_50_ values were calculated as shown in Section “Evaluation of Cytotoxicity against MRC-5 Cells” with the concentration range showed above. Metronidazole (Sigma–Aldrich, MO, USA) was used as positive control.

## Results and Discussion

### Screening of the Kitasato Natural Products Library for CS Inhibitors

*Entamoeba histolytica* has two classes of CS isotypes: EhCS1/EhCS2 and EhCS3. While EhCS1 and EhCS2 are identical except for two amino acid changes (Nozaki et al., 1998), whereas EhCS3 is most divergent from EhCS1 and EhCS2, with 83% amino acid identity (Ali and Nozaki, 2007). Therefore, we selected EhCS1 and EhCS3 as representative CSs in this study.

**FIGURE 2:**
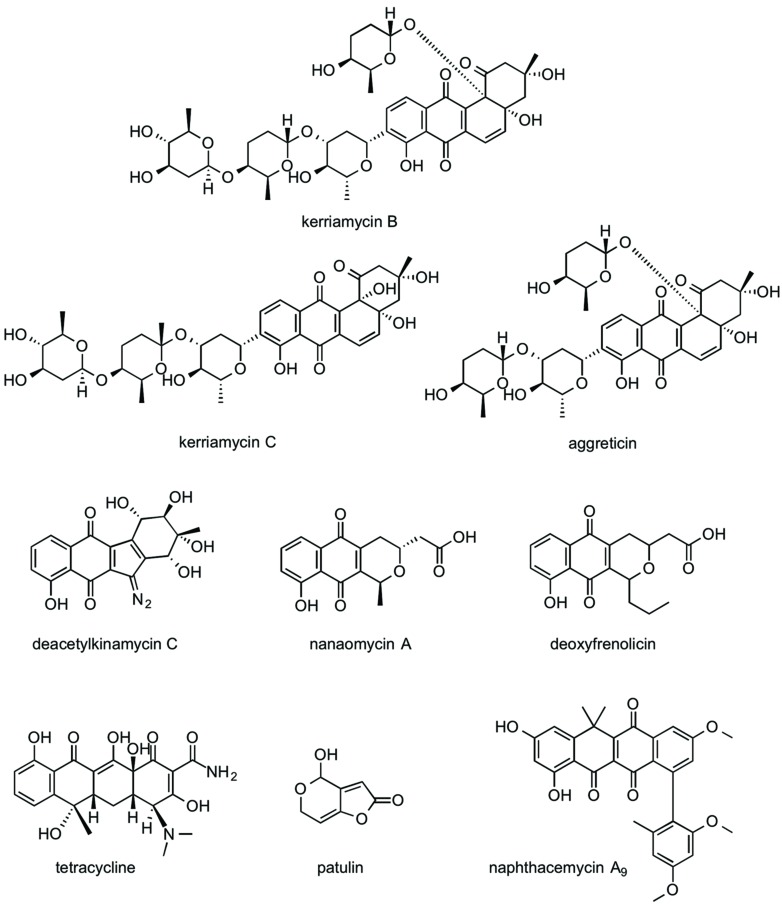
**Structures of EhCS inhibitors from the Kitasato Natural Products Library**.

The Kitasato Natural Products Library consists of >300 natural or semisynthetic compounds. We initially evaluated the EhCS1/EhCS3 inhibitory activity of all compounds at 100 μg/ml. Nine compounds, kerriamycins B and C, aggreticin, tetracycline, patulin, nanaomycin A, deacetylkinamycin C, deoxyfrenolicin, and naphthacemycin A_9_, exhibited inhibition of EhCS1 and/or EhCS3, and their IC_50_ values were subsequently determined (**Table 1**). The structures of these inhibitors are shown in **Figure 2**. Only nephthacemycin A_9_ inhibited EhCS1 selectively, whereas the other compounds showed inhibitory activity against both EhCS1 and EhCS3. Three compounds, kerriamycins B and C, and aggreticin, showed the most potent inhibition (IC_50_ = 0.31–1.2 μM). The IC_50_ values against human fibroblast cells MRC-5 of the nine compounds were also evaluated, as shown in **Table 2**.

**Table 1:** Inhibitory activities of EhCS inhibitors from the Kitasato Natural Products Library.

Compound	IC_50_ (μM)	
	CS1	CS3
Kerriamycin B	0.63 ± 0.07	0.31 ± 0.01
Kerriamycin C	1.2 ± 0.04	0.56 ± 0.04
Aggreticin	0.98 ± 0.05	0.64 ± 0.10
Deacetylkinamycin C	21 ± 2.4	22 ± 3.7
Deoxyfrenolicin	57 ± 2.0	53 ± 1.1
Nanaomycin A	53 ± 3.9	65 ± 4.4
Naphthacemycin A_9_	75 ± 1.8	>490
Tetracycline	170 ± 16	190 ± 9.5
Patulin	490 ± 81	470 ± 37

**Table 2:** *In vitro* anti-amebic activity and cytotoxicity against MRC-5 cells of EhCS inhibitors.

Compound	Cytotoxicity		
	Against *Entamoeba histolytica* IC_50_ (μM)		Against MRC-5 cells IC_50_ (μM)
	Cys (+)	Cys (–)	
Kerriamycin B	26 ± 1.5	23 ± 0.3	21 ± 0.4
Kerriamycin C	6.9 ± 0.4	3.0 ± 0.6	23 ± 1.9
Aggreticin	54 ± 4.8	18 ± 0.7	23 ± 0.6
Deacetylkinamycin C	140 ± 13	18 ± 10	2.3 ± 0.1
Deoxyfrenolicin	1.3 ± 0.04	0.5 ± 0.1	4.7 ± 0.2
Nanaomycin A	12 ± 0.4	0.8 ± 0.04	0.7 ± 0.04
Naphthacemycin A_9_	>390	>390	16 ± 0.6
Tetracycline	>230	>230	430 ± 4.3
Patulin	>650	110 ± 12	110 ± 4.6
Xanthofulvin	>170	>170	NT
Exophillic acid	>140	>140	>100

*NT, Not tested. Cys (+), normal BI-S-33 medium (8 mM L-cysteine); Cys (–), cysteine deprived BI-S-33 medium*.

The naphthoquinone substructure is found in most of the inhibitors shown in **Figure 2**. The most potent EhCS1 and EhCS3 inhibitors, kerriamycins B and C (Hayakawa et al., 1985a,b) and aggreticin (Ōmura et al., 1988) have the same chromophore containing the naphthoquinone substructure. The structural difference among the three compounds is the number of sugar moieties (**Figure 2**). Despite these structural differences, they inhibited EhCSs at similar concentrations, suggesting that the chromophore structure is essential for inhibitory activity but the sugar moieties may not be important. These compounds are known to have biological activities such as anti-Gram-positive bacteria (Hayakawa et al., 1987), anti-cancer properties (Hayakawa et al., 1987), stimulation of platelet aggregation (Ōmura et al., 1988), and inhibition of protein SUMOylation (Fukuda et al., 2009).

Deacetylkinamycin C (kinamycin F) is the perdeacylated compound of kinamycin C, and has the naphthoquinone substructure in its chromophore (**Figure 2**; Ōmura et al., 1973; Gould et al., 1994; Mithani et al., 1994). Its inhibition potency against EhCS ranked next to the kerriamycins, with the IC_50_ values of 21 μM (EhCS1) and 22 μM (EhCS3), respectively. Kinamycin C displays anti-Gram-positive bacterial activity and anti-tumor activity (Ōmura et al., 1973; Hasinoff et al., 2006). Kinamycins can bind to DNA weakly and produce DNA- and protein-damaging effects (Hasinoff et al., 2006; O’Hara et al., 2007).

Nanaomycin A, a naphthoquinone antibiotic, has anti-malarial activity (Tanaka et al., 1999) along with anti-fungal, anti-bacterial, and anti-mycoplasma activities (Tanaka et al., 1975). Both nanaomycin A and its structural-related compound, deoxyfrenolicin (Ellestad et al., 1966; Iwai et al., 1978), showed modest inhibitory activity against EhCS1 and EhCS3 (IC_50_ = 53–65 μM). The structural difference between the two compounds is the length of alkyl chain attached to a chromophore. Therefore, the chromophore containing the naphthoquinone substructure must be important for the inhibition of EhCSs. Unfortunately, cytotoxicities against MRC-5 cells of nanaomycin A and deoxyfrenolicin were also more potent than CS inhibitory activities, as in the case of deacetylkinamycin C.

Tetracycline also has a naphthoquinone-like substructure in its chromophore. However, inhibition of EhCS1 and EhCS3 by tetracycline was weak, with IC_50_ values of 170 and 190 μM, respectively. One of the mycotoxins, patulin, is the smallest inhibitor found in this screening. Inhibition of both EhCS1 and EhCS3 by patulin was also weak.

Naphthacemycin A_9_, isolated from a broth of *Streptomyces* sp. KB3346-5 was found to be a novel potentiator of imipenem activity against methicillin-resistant *Staphylococcus aureus* (Ōmura et al., 2009). Naphthacemycins have naphthacene structures with an aryl group attached via biaryl bond. This compound showed selective inhibitory activity against EhCS1; the IC_50_ value was 75 μM. However, it displayed no inhibition of EhCS3.

Taken together from the results of screening of the Kitasato Natural Products Library, the naphthoquinone moiety appears to be a common important structure for the inhibition of EhCSs.

### Screening of the Microbial Broths for CS Inhibitors

Extracts of 9,173 fungal and actinomycete broths were screened for inhibition of CS activity of recombinant EhCS1 and EhCS3. Screening results are shown in **Table 3**. Many microbial broth extracts selectively inhibited one isoenzyme, but not the other. We found that 104 extracts solely inhibited EhCS1, while 167 extracts solely inhibited EhCS3. Seventy extracts inhibited both EhCS1 and EhCS3. We found a comparable number of active extracts from broths of fungi and actinomycetes. This is in good contrast to the CS inhibitors found from the Kitasato Natural Products Library; most of them inhibited both EhCS1 and EhCS3.

**Table 3:** EhCS inhibitors found in microbial broth extracts.

Origin	Number of samples	CS1	CS3	CS1 and CS3
Fungi	4,800	64	87	47
Actinomycetes	4,373	40	80	23
**Total**	9,173	104	167	70
		(1.1%)	(1.8%)	(0.8%)

We tested the above-mentioned active broth extracts showing CS inhibitory activity for cytotoxicity using human fibroblast cells (MRC-5), and selected two fungal broth extracts with no or low cytotoxicity. One extract was obtained from *Penicillium* sp. if08054, which showed inhibitory activity against both isotypes. The other extract was from *Exophiala* sp. FKI-7082, which showed inhibitory activity specifically against EhCS1. We isolated xanthofulvin and exophillic acid as the active components from the broths of these two fungal strains, respectively.

### Xanthofulvin, an EhCS1 and EhCS3 Inhibitor from a Broth of *Penicillium* sp. if08054

**FIGURE 3:**
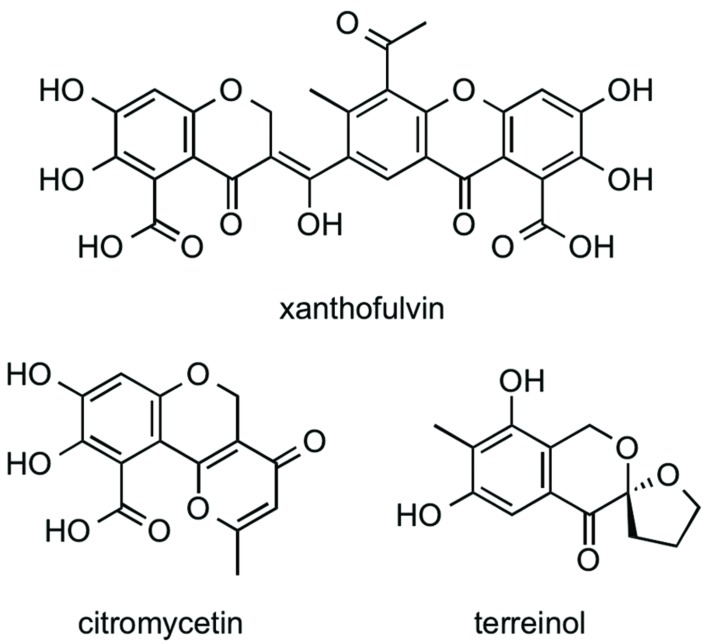
**Structures of xanthofulvin, citromycetin, and terreinol**.

Xanthofulvin was isolated from the *n*-BuOH extract of a broth of *Penicillium* sp. if08054 using an ODS column. CS inhibitory activity was found in the 40% acetonitrile (MeCN) fraction. Subsequently, about 2 mg of xanthofulvin (**Figure 3**; Kumagai et al., 2003) was purified by preparative HPLC from 50 ml of the cultured broth. Two structurally related compounds, citromycetin (Robertson et al., 1951; Capon et al., 2007) and terreinol (Macedo et al., 2004) were also isolated from the 20% MeCN fraction and 40% MeCN fraction, respectively. The structures of the compounds were confirmed by 1D and 2D NMR spectra and MS spectra. Xanthofulvin was originally isolated as a semaphorin inhibitor with the IC_50_ value of 0.09 μg/mL (Kumagai et al., 2003). We compared CS inhibitory activity of xanthofulvin, citromycetin, and terreinol. The IC_50_ values of these compounds against two EhCS isotypes are shown in **Table 4**. Among these three compounds, only xanthofulvin inhibited EhCSs, whereas citromycetin and terreinol showed no inhibitory activities against EhCSs at 1,400 or 1,600 μM. Xanthofulvin inhibited EhCS1 more potently than EhCS3: the IC_50_ values against EhCS1 and EhCS3 are 7.9 and 110 μM, respectively. Xanthofulvin has a naphthoquinone-like substructure similar to EhCS inhibitors identified from the Kitasato Natural Products Library. Although citromycetin and terreinol also have xanthofulvin-related structures, no/weak inhibitory activities against EhCSs were detected. Therefore, the three-dimensional conformation provided by the dimeric-like structure of xanthofulvin seems to be important for producing inhibition of EhCSs.

**Table 4:** Inhibitory activities of xanthofulvin, citromycetin, and terreinol against EhCS1 and EhCS3.

Compound	IC_50_ (μM)	
	CS1	CS3
Xanthofulvin	7.9 ± 1.3	110 ± 8.2
Terreinol	>1,600	>1,600
Citromycetin	>1,400	>1,400

### Exophillic Acid, a CS1 Inhibitor from a Broth of *Exophiala* sp. FKI-7082

**FIGURE 4:**
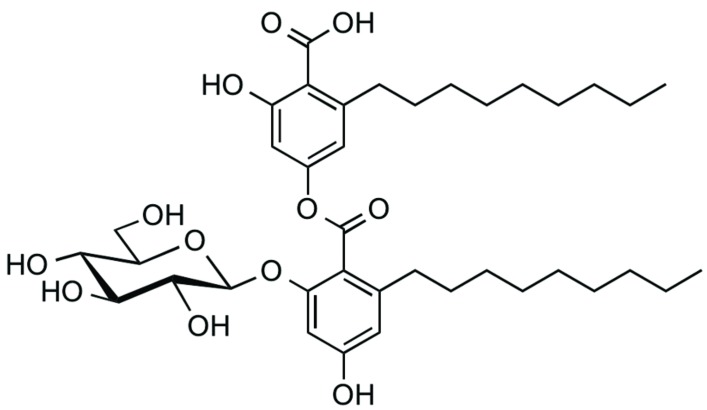
**Structure of exophillic acid**.

Exophillic acid was isolated from the ethyl acetate extract of the broth of *Exophiala* sp. FKI-7082 by silica gel and ODS column chromatography (**Figure 4**; Ondeyka et al., 2003). The structure was clarified by measurement of MS and NMR spectra. Exophillic acid was originally isolated from a broth of *Exophiala pisciphila*, and its main biological activity includes inhibition of the strand transfer reaction of HIV-1 integrase (Ondeyka et al., 2003). Exophillic acid inhibited EhCS1 with the IC_50_ value of 24 ± 2.9 μM, whereas it showed no inhibition against EhCS3 even at a concentration of 2.5 mM. Exophillic acid is a dimeric alkyl-benzoate glucoside and does not contain naphthoquinone. Exophillic acid likely recognizes the structural differences between the two enzymes. Further structure elucidation is needed to understand EhCS1-specific inhibition by exophillic acid.

### Anti-*Entamoeba histolytica* Activity of CS Inhibitors

We next examined whether the CS inhibitors show an amebicidal activity against *E. histolytica* trophozoites *in vitro*. To verify whether an amebicidal activity is due to inhibition of CS, we estimated the IC_50_ values of inhibitors against *E. histolytica* trophozoites in the cysteine-supplemented and cysteine-deprived BI-S-33 medium. In theory, the amebicidal activity and the growth inhibitory effects should be more pronounced in the absence of cysteine than in the presence of cysteine if these effects are due to the inhibition of CS. The amebicidal activities of CS inhibitors are shown in **Table 4**, together with the cytotoxicity of the compounds against MRC-5 cells.

Deacetylkinamycin C, nanaomycin A, and patulin showed cysteine-dependent amebicidal activity. The IC_50_ of deacetylkinamycin C against EhCSs (21 and 22 μM) and the IC_50_ value in the cysteine-deprived medium (18 μM) were comparable, which suggests the amebicidal effect of deacetylkinamycin C is likely due to CS inhibition. Nanaomycin A exhibited amebicidal activity 15-fold more potent in cysteine-deprived medium, however, the IC_50_ values against EhCSs were less potent than the amebicidal activity. Though patulin also showed selective amebicidal activity, inhibition of EhCSs was less potent than its anti-amebic activity. Xanthofulvin and exophillic acid did not show amebicidal activities at 170 and 140 μM, respectively. It was reported that xanthofulvin was not likely incorporated into cells (Kaneko et al., 2006; Kikuchi et al., 2009). In an *E. histolytica* case, it may be difficult to incorporate this compound into cells. The reason why exophillic acid did not exhibit amebicidal activity was unknown.

Deacetylkinamycin C, which has DNA- and protein-damaging effects (Colis et al., 2014), showed potent cytotoxicity against MRC-5 cells; the IC_50_ value against MRC-5 cells was lower than those of the IC_50_ values against *E. histolytica* cells and against EhCS. Nanaomycin A and deoxyfrenolicin also showed cytotoxicity against MRC-5 cells, at the lower concentrations showing CS inhibition, as in the case of deacetylkinamycin C. Although most compounds that inhibited EhCS in this study were toxic against a human-derived cell line, MRC-5, it is possible to design and produce by organic synthesis their derivatives that possess EhCS-dependent anti-amebic activity but lack cytotoxicity to humans. To design such derivatives, structural studies are needed to determine the moieties required for binding to EhCS.

In this study, we identified nine general EhCS inhibitors and two isotype-specific EhCS inhibitors, naphthacemycin A_9_ and exophillic acid, from microbial secondary metabolite sources. A naphthoquinone structure seemed to contribute significantly to EhCS inhibition. One EhCS1-selective inhibitor, naphthacemycin A_9_, has a naphthacene structure with an aryl group attached via biaryl bond. The other EhCS1-selective inhibitor, exophillic acid, has a depside structure which two aryl groups attach to via an ester bond. For EhCS1-selective inhibition, the substituted small aryl group is likely important. Further structural studies using EhCS inhibitors found in this study are needed for developing anti-amebic drugs. We also found EhCS3-selective inhibitors from both fungal and actinomycete broths. These EhCS3-selective inhibitors will be purified and identified in future studies. Since CS is also present in other protozoa, such as *Trichomonas vaginalis*, *Leishmania major*, and *Trypanosoma cruzi*, the newly identified CS inhibitors in this study can also be exploited to develop drugs against other neglected parasitic diseases.

## Conflict of Interest Statement

The authors declare that the research was conducted in the absence of any commercial or financial relationships that could be construed as a potential conflict of interest.
